# Realization of a scanning soft X-ray microscope for magnetic imaging under high magnetic fields

**DOI:** 10.1107/S1600577518009177

**Published:** 2018-08-02

**Authors:** Yoshinori Kotani, Yasunori Senba, Kentaro Toyoki, David Billington, Hiroyuki Okazaki, Akira Yasui, Wakana Ueno, Haruhiko Ohashi, Satoshi Hirosawa, Yu Shiratsuchi, Tetsuya Nakamura

**Affiliations:** a Japan Synchrotron Radiation Research Institute (JASRI), SPring-8, Sayo 679-5198, Japan; b Elements Strategy Initiative Center for Magnetic Materials (ESICMM), National Institute for Materials Science, Tsukuba 305-0047, Japan; c Department of Materials Science and Engineering, Graduate School of Engineering, Osaka University, Suita 565-0871, Japan

**Keywords:** X-ray microscopy, magnetic domains, X-ray magnetic circular dichroism, Nd–Fe–B

## Abstract

For the purpose of imaging element- and shell-specific magnetic distributions under high magnetic fields, a scanning soft X-ray microscope has been developed at beamline BL25SU, SPring-8, Japan. The scanning X-ray microscope utilizes total electron yield detection of absorbed circularly polarized soft X-rays in order to observe magnetic domains through the X-ray magnetic circular dichroism effect. Crucially, this system is equipped with an 8 T superconducting magnet. The performance and features of the present system are demonstrated by magnetic domain observations of the fractured surface of a Nd_14.0_Fe_79.7_Cu_0.1_B_6.2_ sintered magnet.

## Abstract 2

A scanning soft X-ray microscope for magnetic imaging using an 8 T superconducting magnet, installed at BL25SU, SPring-8, is described. With the instrument the magnetic domain structure of an Nd–Fe–B sintered magnet is demonstrated.

## 1. Introduction   

One of the most useful ways to assess how the reversed magnetic domains are generated and evolve during magnetization reversal processes is by directly observing the magnetic domain structure. Currently, X-ray magnetic circular dichroism (XMCD) microscopy is the only practical technique for imaging element- and shell-specific magnetic distributions (Schütz *et al.*, 1987 ▸; Chen *et al.*, 1990 ▸; Stöhr *et al.*, 1993 ▸; Fischer & Ohldag, 2015 ▸; Fischer, 2017 ▸). In recent years, this technique has seen a surge in popularity and there is a growing demand for its application under high magnetic fields, especially for investigating highly anisotropic magnetic materials. However, XMCD imaging under high magnetic fields (larger than 2 T) requires the use of a superconducting magnet (SCM). Combining the XMCD imaging technique with a SCM has proven to be a demanding technical challenge because of the practical geometric restrictions in placing the sample, focusing optics and translation stages in the field without interference from the superconducting coils and their cooling system. Engineering a solution to this requires radically different equipment design.

A particularly challenging material for magnetic domain observations has been magnetically anisotropic Nd–Fe–B sintered magnets. In these materials, from both physical and metallurgical points of view, magnetic domain observations throughout the entire magnetization process are required to help elucidate the coercivity mechanism (Hirosawa, 2015 ▸; Hono & Sepehri-Amin, 2012 ▸; Coey, 2012 ▸; Gutfleisch *et al.*, 2011 ▸). Since the coercivity of commercial Nd–Fe–B sintered magnets is about 1.2 T, it is necessary to apply magnetic fields larger than 3 T in order to saturate the magnetization. Moreover, a magnetic field greater than 5 T is desirable so that permanent magnets with coercivities greater than 3 T can become the targets of future investigations. However, the maximum magnetic field used with soft X-ray microscopy instruments that has ever been reported does not exceed 0.5 T (Kim *et al.*, 2006 ▸; Im *et al.*, 2003 ▸). To date, Kerr microscopy (Takezawa *et al.*, 2014 ▸; Khlopkov *et al.*, 2004 ▸), magnetic force microscopy (MFM) (Yamaoka *et al.*, 2014 ▸), scanning transmission X-ray microscopy (STXM) with XMCD (Ono *et al.*, 2011 ▸; Ohtori *et al.*, 2014 ▸), and photoemission electron microscopy (PEEM) with XMCD (Yamamoto *et al.*, 2008 ▸; Yamaguchi *et al.*, 2011 ▸) have been applied as methods for observing magnetic domains in Nd–Fe–B magnets, but these observations have been limited to polished surfaces or transmittable thin films, in which the coercivity significantly decreases compared with the bulk value (Hirosawa *et al.*, 1987 ▸; Fukagawa & Hirosawa, 2008 ▸). This suggests that the polished surfaces and thin films are not representative of the internal magnetic state. On the other hand, it has been observed that fractured surfaces are much better at maintaining their coercivity because they do not lose the grain boundary phase during the fracturing procedure (Nakamura *et al.*, 2014 ▸). The fractured surface is, therefore, a much more favorable target for observing magnetic domains in order to help elucidate the coercivity mechanism.

Unfortunately, there are few methods for observing magnetic domain structures in fractured surfaces. Since the surface roughness of these materials is approximately proportional to the grain size, the distance between the top and bottom of the surface may average 5 µm in standard Nd–Fe–B sintered magnets. It is, therefore, difficult to image the magnetic domains in the fractured surface with MFM because the probing needle cannot easily follow along the irregular surface. Moreover, observations of fractured surfaces under high magnetic fields are especially difficult; a spin-polarized scanning electron microscope (SP-SEM) has successfully observed magnetic domains in the fractured surface (Kohashi *et al.*, 2014 ▸), however, magnetic fields cannot be applied due to the electron microscopy lenses (PEEM also suffers from this problem). Although PEEM and SP-SEM are not applicable to magnetic domain observations under magnetic fields, a scanning X-ray microscope (SXM) using a focused X-ray beam would make these observations possible: SXMs are capable of observing uneven surfaces within the focal depth (axial distance from the focal point over which imaged objects are acceptably sharp) because the working distance between the lens and the sample surface is in the range of millimetres. Furthermore, by composing the instruments of the SXM (including not only the lens but also the scanning and positioning units) from non-magnetic devices, the application of large magnetic fields is permitted. In this report, a SXM apparatus equipped with an 8 T SCM is presented as a new tool for investigating the magnetic domain structures in inhomogeneous magnetic materials under high magnetic fields. In order to demonstrate the capability of this system, the magnetic domain structure in the fractured surface of an Nd_14.0_Fe_79.7_Cu_0.1_B_6.2_ sintered magnet is imaged using soft XMCD at applied magnetic fields of 0 T (thermally demagnetized state) and ±8 T (fully saturated states).

## 2. Results and discussion   

**Figure 1:**
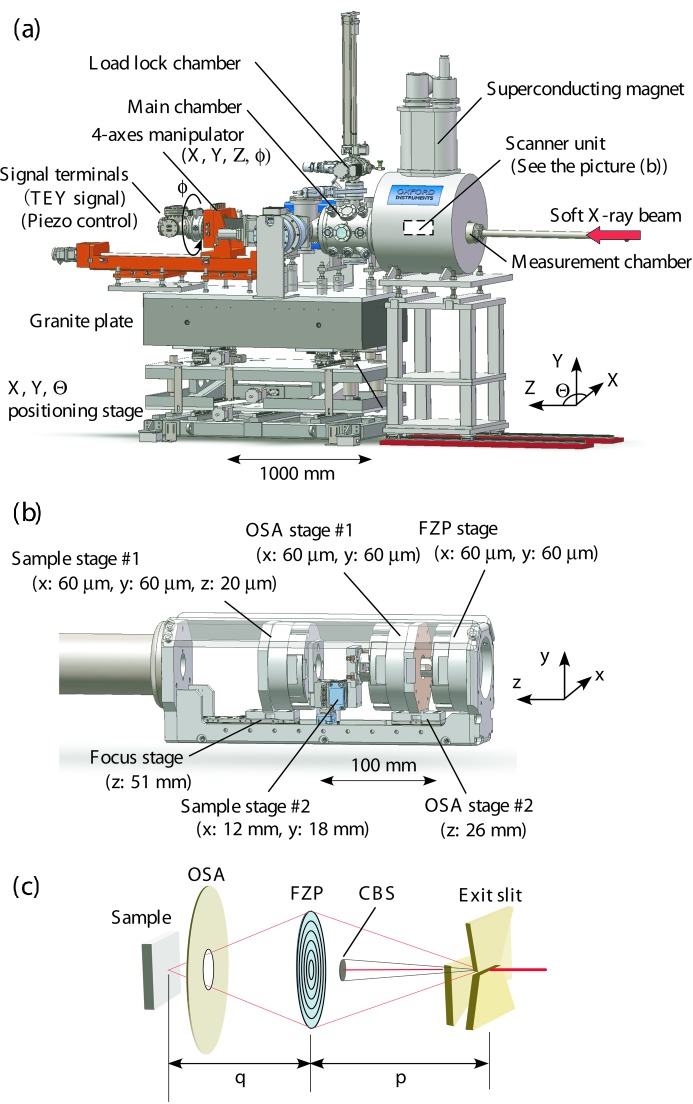
Schematic drawings of (*a*) the SXM apparatus equipped with an 8 T superconducting magnet and (*b*) the scanner unit composed of the FZP, OSA and the sample stages. The values in parentheses indicate the maximum travel lengths for the piezo actuator stages. (*c*) Schematic drawing of the layout of optical components. Detailed specifications of each the component are given in the text.

The high-magnetic-field SXM technique was developed at the soft X-ray beamline BL25SU of the SPring-8 synchrotron, Japan (Senba *et al.*, 2016 ▸; Hara *et al.*, 2003 ▸). The SXM apparatus is shown in Fig. 1(*a*) ▸ and consists of an ultra-high-vacuum (UHV) chamber installed on a positioning stage with a stable granite plate that is used for alignment of the chamber along the X-ray beam axis. An 8 T SCM with a bore diameter of 149 mm (manufactured by Oxford instruments Ltd, UK) was installed on guide rails in the floor. Fig. 1(*b*) ▸ shows the scanning unit which is equipped with seven short-travel-distance (∼10 µm) piezo actuator-drive units that mount the Fresnel zone plate (FZP), order-sorting aperture (OSA) and the sample. The piezo actuator stages for sample stage #1, OSA stage #1 and the FZP stage in Fig. 1(*b*) ▸ are from nPoint Inc., model numbers NPXY60Z20-257, NPXY60-258 and NPXY60-258, respectively, and are all non-magnetic and equipped with digital encoders. The scanner unit is supported by a titanium pipe that is connected to a four-axis motorized stage for adjusting the position of the scanning unit relative to the beam. Four additional piezo stages with longer travel distances (∼10 mm) are used for coarse alignment of the sample position and for adjusting the focusing distance between the FZP, OSA and the sample. These four piezo stages are located at sample stage #2, OSA stage #2, and the focus stage in Fig. 1(*b*) ▸ and are from MICRONIX USA, LLC, model number PPS-20, and are all non-magnetic nano-positioning stages of the inertial drive-type with digital encoders. In the present development, the beam-spot size of about 100 nm is designed so that the focal depth is obtained over a range of ±5 µm from the focal point along the beam axis.

Fig. 1(*c*) ▸ shows the focusing optics adopted in the present development. The FZP was nano-fabricated from a 200 nm-thick Ta film deposited on a SiC membrane substrate which has a 200 nm-thick window. The radius, *r*, and outermost zone width of the FZP, 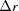, are 155 µm and 40 nm, respectively. The pinhole diameter of the OSA is 50 µm and was fabricated using a Pt plate with 0.2 mm thickness. The diameter of the central beam stopper (CBS) is 100 µm and is made of Au which was deposited on a SiC membrane. The opening size of the exit slit of the beamline monochromator was assumed to be the size of the light source, σ, in the present focusing optics. The SXM apparatus was installed in a position such that the distance between the FZP and the exit slit, *p*, is 12.0 m. The focal length, *q*, of the FZP was designed to be 10 mm at an incident soft X-ray beam energy, *E*, of 1000 eV. The size of the focused beam, δ, which is defined by the full width at half-maximum (FWHM), is estimated as follows (Günther *et al.*, 2002 ▸),
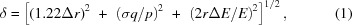
where *E* is the incident X-ray energy and 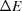 is the FWHM energy resolution. According to (1), the design δ was deduced to be 73 nm for 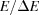 = 9000 and σ = 50 µm. Note that the value of 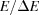 is used in the following discussion because it is more popular in soft X-ray optics than 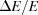 used in (1). The degree of circular polarization of the incident beam was estimated to be 0.96 at 400 eV in a previous report (Hirono *et al.*, 2005 ▸), and is almost the same in the energy regions of the Fe *L*_3_- (707.9 eV) and Nd *M*_4_-edges (1000.4 eV).

A magnetically anisotropic Nd–Fe–B sintered magnet with composition Nd_14.0_Fe_79.7_Cu_0.1_B_6.2_ was prepared using the standard strip-casting, jet-milling, magnet compaction and sintering process. After sintering at 1020°C, the sample was annealed at 540°C for 2 h in order to increase the coercivity to about 1.0 T. This sample was a piece cut from a larger block which was used in a previous study (Nakamura *et al.*, 2014 ▸). The sample was rod-shaped with the long axis parallel to both the easy magnetization direction (*c*-axis of Nd_2_Fe_14_B) and the soft X-ray beam. In order to prevent oxidization of the fractured surface, the sample was fractured in the UHV chamber of the XMCD apparatus where the vacuum level was below 5 × 10^−7^ Pa. The Fe *L*_3_- (707.9 eV) and Nd *M*_4_-edges (1000.4 eV) were used to obtain element-specific SXM images for Fe and Nd, respectively. The XMCD signal is given by 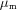 = 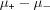, where 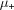 and 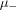 represent absorption of circularly polarized soft X-ray photons with positive and negative helicity, 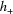 and 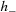, respectively. The absorption signal was recorded by means of the total electron yield (TEY) method. When recording the TEY signal, a retarding bias voltage of −18 V was applied to the sample, while the OSA was grounded.

**Figure 2:**
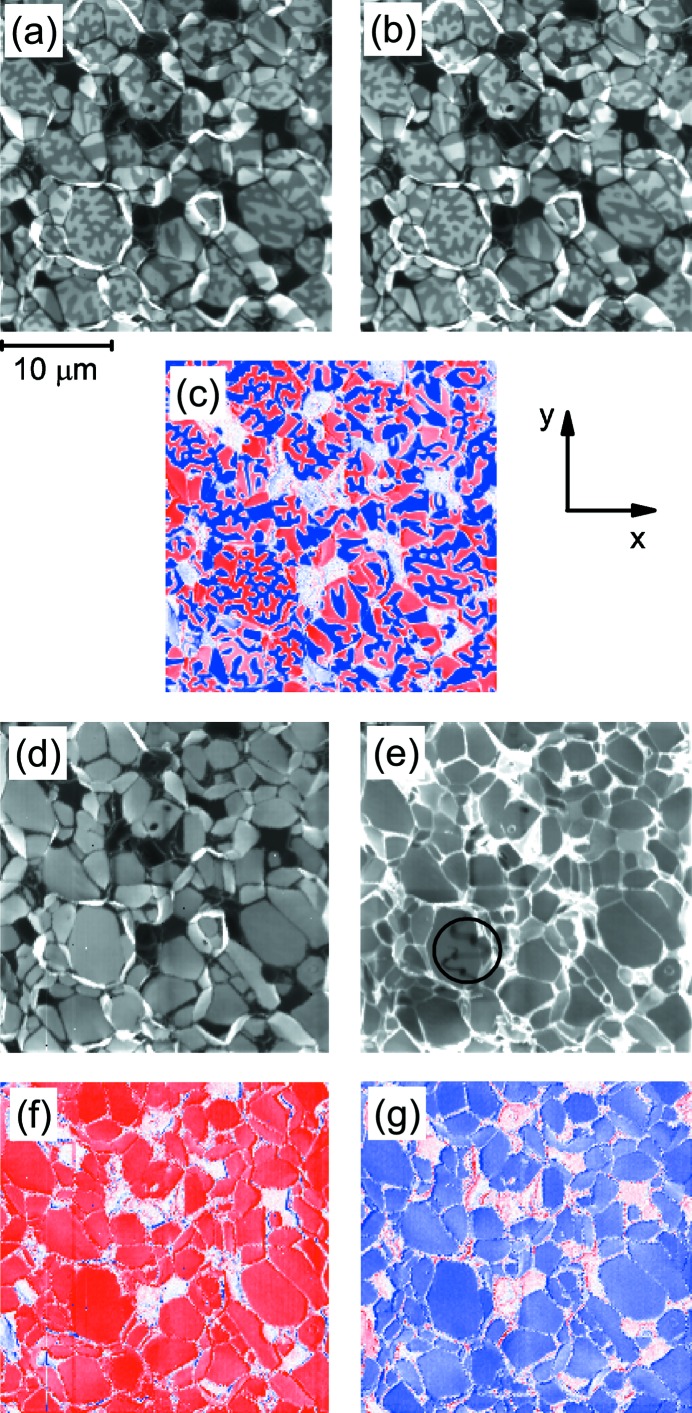
Panels (*a*) and (*b*) show the Fe *L*_3_-edge X-ray absorption images for 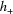 and 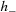, respectively. The Fe *L*_3_-edge XMCD image obtained from the subtraction of (*a*) and (*b*) is shown in (*c*). Panels (*d*) and (*e*) show the helicity-averaged X-ray absorption images at the Fe *L*_3_- and Nd *M*_4_-edges, respectively. Panels (*f*) and (*g*) show the Fe *L*_3_-edge XMCD images at applied fields of +8 T and −8 T, respectively. Red and blue colors in the XMCD images indicate that 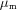 is positive and negative, respectively. The *x*- and *y*-directions coincide with those in Fig. 1 ▸. The XMCD images in panels (*c*), (*f*) and (*g*) have been normalized by their respective helicity-averaged X-ray absorption images.

Figs. 2(*a*) and 2(*b*) ▸ show the Fe *L*_3_-edge X-ray absorption images, 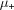 and 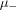, recorded for 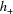 and 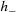, respectively, of the fractured surface of the sintered Nd_14.0_Fe_79.7_Cu_0.1_B_6.2_ magnet in its thermally demagnetized state. The magnetic domain contrast superposed on the texture of the fractured surface is clearly observed due to the large XMCD effect at the Fe *L*_3_-edge. Fig. 2(*c*) ▸ shows the difference image between Figs. 2(*a*) and 2(*b*) ▸. This gives the net XMCD contrast, 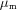, corresponding to the magnetic domain structure. Here, the magnetization direction is parallel and antiparallel to the wavevector of the soft X-ray beam for red and blue colors, respectively. Fig. 2(*d*) ▸ shows the helicity-averaged image [obtained by 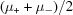] at the Fe *L*_3_-edge. Here, the edges of the grains are brighter because the footprint of the beam spot becomes larger when the surface normal is not parallel to the incident X-ray beam and thus increases the TEY signal. The dark (almost black) contrast in Figs. 2(*a*), 2(*b*) and 2(*d*) ▸ corresponds to the Nd-rich phase with much lower Fe concentration. This is evidently confirmed by the bright (almost white) contrast in Fig. 2(*e*) ▸ which shows the helicity-averaged Nd *M*_4_-edge absorption image, and also by the lack of Fe *L*_3_-edge XMCD signal indicated by the almost white contrast in Fig. 2(*c*) ▸. Note that the darker area with a curved-line shape marked by the circle in Fig. 2(*e*) ▸ has been contaminated by carbon due to soft X-ray irradiation for a long time during some tests before measuring the Nd *M*_4_-edge. The reduction of the TEY signal due to the carbon contamination is estimated to be about 19% in Fig. 2(*e*) ▸. In a separate experiment, we estimated that the reduction of the TEY signal was less than about 1% for each image with an irradiation time of a few milliseconds for each measurement point, although this reduction is not linear with time. Figs. 2(*f*) and 2(*g*) ▸ show the Fe *L*_3_-edge XMCD images under applied magnetic fields of +8 T and −8 T, respectively, where the magnetization is saturated along the magnetic field direction. The XMCD imaging is successful even when high magnetic fields are applied to the sample. The small decrease of the absolute values of XMCD in Figs. 2(*f*) and 2(*g*) ▸ compared with those in Fig. 2(*c*) ▸ originates from the external magnetic fields affecting the TEY efficiency (Goering *et al.*, 2000 ▸). Note that the blue areas in Fig. 2(*f*) ▸ and red areas in Fig. 2(*g*) ▸ are only located at the steep grain edges and are caused by a small drift of the sample position. The magnetic fields also cause a significant shift of the entire scanner unit from the initial position by about 0.8 mm when 8 T is applied, but the shift of the position can be easily adjusted using the four-axes manipulator depicted in Fig. 1(*a*) ▸. The reason for the shift is because the scanner unit has some weakly paramagnetic parts. In fact, the influence of the shift to the X-ray absorption imaging is very small because the entire scanning unit (including the focusing optics and sample stage) move together as one. The relationship between the sample, the OSA and the FZP is almost unaffected within an accuracy of about 1 µm, presumably because the sample is ferromagnetic.

The focused beam sizes corresponding to the spatial resolution of the images were evaluated using the magnetic domain contrast in the Nd–Fe–B sintered magnet. This method works well since the expected domain wall width is about 5 nm (Park *et al.*, 2000 ▸) which is much smaller than the beam size in the present design. In practice, the beam shape is anisotropic because the exit-slit opening is rectangular, so we have to determine 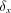 and 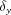 independently. Furthermore, small differences in the divergence angle of the incident beam at the exit-slit in the *x*- and *y*-directions also contribute to the asymmetric shape of the focused beam, leading to small deviations from the values estimated from equation (1). To determine the best possible spatial resolution in the *x*-direction, 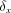 (*y*-direction, 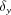), line scans were measured across domain walls running approximately perpendicular to the scan direction as a function of slit width, 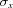 (height, 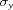), with the slit height (width) held constant at 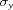 = 150 µm (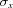 = 400 µm) in order to prevent the photon flux from becoming too low (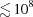
 photon s^−1^) at small slit sizes. The best spatial resolutions obtained at the Fe *L*_3_-edge were 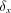 = 90 nm when 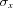 = 24 µm and 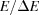 = 3000 giving a photon flux of 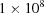
 photon s^−1^, and 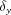 = 72 nm when 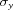 = 49 µm and 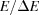 = 9000 giving a photon flux of 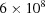
 photon s^−1^, where the photon flux was determined by a PIN-type photodiode detector (note that the energy resolution only depends on the slit height, 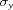, due to the geometry of BL25SU). These values are reasonably consistent with what is expected from equation (1). In order to increase the photon flux and, hence, improve the statistics, we chose 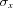 = 800 µm, 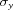 = 300 µm for the measurements presented in Fig. 2 ▸. This gave 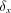 = 224 nm, 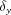 = 127 nm, 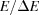 = 1500 and a photon flux of about 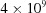
 photon s^−1^, resulting in a TEY current of the order of 10 pA. The spatial resolution is typically degraded by about a factor of 1.6 at an axial distance of ±5 µm from the focal point.

**Figure 3:**
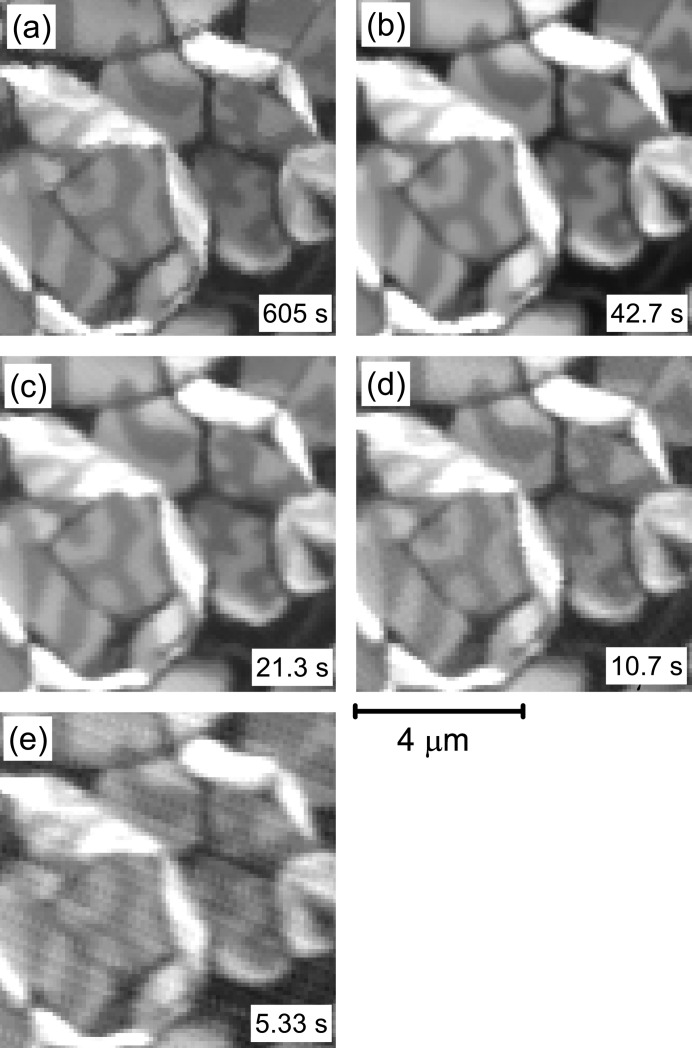
Fe *L*_3_-edge X-ray absorption images recorded using various scanning modes. The conditions for the spatial resolution and the photon flux of the focused beam are identical to those in Fig. 2 ▸. (*a*) Image obtained by the step scan method with a 100 nm step, where the signal averaging time, τ, for each pixel of the image is 3.3 ms. Panels (*b*), (*c*), (*d*) and (*e*) were obtained using the quick scan mode with 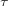 = 3.3 ms, 1.7 ms, 0.83 ms and 0.42 ms, respectively. The corresponding acquisition times for an 8 µm × 8 µm area are represented in each image and are converted from those for the full 60 µm × 60 µm area.

Fig. 3 ▸ shows Fe *L*_3_-edge X-ray absorption images recorded with various scan modes and speeds. In addition to the conventional step scan mode in Fig. 3(*a*) ▸, a quick scan mode is examined in Figs. 3(*b*)–3(*e*) ▸. In the quick scan mode, sample stage #1 in Fig. 1(*b*) ▸ is continuously scanned along the *x*-axis with a 250 kHz sampling rate of the TEY signal. Despite the much longer acquisition time for the step scan mode in Fig. 3(*a*), the image quality in Fig. 3(*b*) is much improved, even though the signal averaging times are almost the same between Figs. 3(*a*) and 3(*b*) ▸. The reduced image quality in the step scan mode is possibly caused by vibration of the sample due to the frequent acceleration and deceleration at each measured position. The acquisition time for the 60 µm × 60 µm area is evaluated as 40 min, 20 min, 10 min and 5 min for the scan modes used in Figs. 3(*b*), 3(*c*), 3(*d*) and 3(*e*) ▸, respectively. The times denoted in Figs. 3(*b*)–3(*e*) ▸ have been scaled from the measurement time for a 60 µm × 60 µm area to the selected 8 µm × 8 µm area. The image quality depends reasonably on the scan speed in Figs. 3(*b*)–3(*d*) ▸ and becomes clearly worse in Fig. 3(*e*) ▸. The scan speed of Fig. 3(*e*) ▸ is, however, still available for test scans to determine the image area.

## 3. Conclusions   

In summary, we have developed a scanning soft X-ray microscopy instrument equipped with an 8 T superconducting magnet for the purpose of measuring element- and shell-specific magnetic distributions in inhomogeneous magnetic systems under high magnetic fields. To demonstrate the instruments capabilities, we have successfully imaged the magnetic domains in the fractured surface of a Nd–Fe–B sintered magnet in the thermally demagnetized (0 T) and fully saturated (±8 T) states, and we have mapped elemental distributions by determining the helicity-averaged absorption at both the Fe *L*_3_- and Nd *M*_4_-edges. We have demonstrated different scan and data acquisition modes and found that a quick scan mode (in which the sample stage is continuously scanned along the *x*-axis) produces higher quality images in much faster times than the step scan mode. Finally, we plan to develop the new instrument further in the near future through measurements of XMCD spectra at fixed positions, and we also plan to install a cryostat in order to access larger regions of phase diagrams through measurements at both low temperatures and high magnetic fields.
